# Nanoscopic quantification of sub-mitochondrial morphology, mitophagy and mitochondrial dynamics in living cells derived from patients with mitochondrial diseases

**DOI:** 10.1186/s12951-021-00882-9

**Published:** 2021-05-13

**Authors:** Weiwei Zou, Qixin Chen, Jesse Slone, Li Yang, Xiaoting Lou, Jiajie Diao, Taosheng Huang

**Affiliations:** 1 Division of Human Genetics, Cincinnati Children’s Hospital Medical Center, 3333 Burnet Avenue, Cincinnati, OH 45229 USA; 2 Department of Obstetrics and Gynecology, Reproductive Medicine Center, The First Affiliated Hospital of Anhui Medical University, Hefei, 230022 China; 3 Department of Cancer Biology, University of Cincinnati College of Medicine, Cincinnati, OH 45267 USA; 4 Institute of Materia Medica, Shandong First Medical University & Shandong Academy of Medical Sciences, Jinan, 250062 China; 5 Present Address: Department of Pediatrics, Jacobs School of Medicine and Biomedical Sciences, University at Buffalo, Buffalo, NY 14203 USA; 6 Department of Pediatrics, Xiangya Hospital, Central South University, Changsha, 410008 Hunan China; 7 School of Laboratory Medicine and Life sciences, Wenzhou Medical University, Wenzhou, 325035 Zhejiang China

**Keywords:** Nanoscope, Mitochondrial disease, SLC25A46, Cristae, Mitophagy

## Abstract

*SLC25A46* mutations have been found to lead to mitochondrial hyper-fusion and reduced mitochondrial respiratory function, which results in optic atrophy, cerebellar atrophy, and other clinical symptoms of mitochondrial disease. However, it is generally believed that mitochondrial fusion is attributable to increased mitochondrial oxidative phosphorylation (OXPHOS), which is inconsistent with the decreased OXPHOS of highly-fused mitochondria observed in previous studies. In this paper, we have used the live-cell nanoscope to observe and quantify the structure of mitochondrial cristae, and the behavior of mitochondria and lysosomes in patient-derived *SLC25A46* mutant fibroblasts. The results show that the cristae have been markedly damaged in the mutant fibroblasts, but there is no corresponding increase in mitophagy. This study suggests that severely damaged mitochondrial cristae might be the predominant cause of reduced OXPHOS in *SLC25A46* mutant fibroblasts. This study demonstrates the utility of nanoscope-based imaging for realizing the sub-mitochondrial morphology, mitophagy and mitochondrial dynamics in living cells, which may be particularly valuable for the quick evaluation of pathogenesis of mitochondrial morphological abnormalities.

**Supplementary Information:**

The online version contains supplementary material available at 10.1186/s12951-021-00882-9.

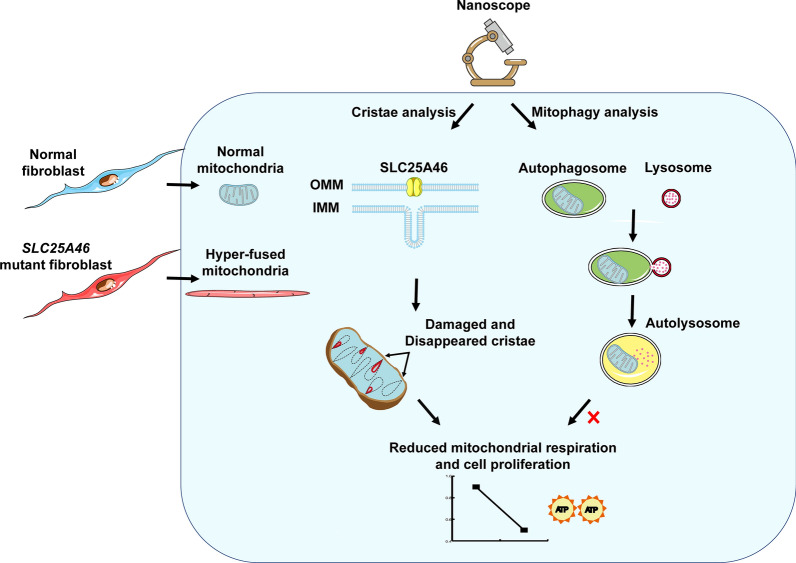

## Background

The mitochondrion is the cellular organelle which is critical for energy metabolism in mammals and most other eukaryotes. Mitochondrial dysfunction caused by nuclear DNA (nDNA) or mitochondrial DNA (mtDNA) defects lead to cellular respiratory chain and energy metabolism disorders, resulting in a group of multi-system diseases [1, 2]. A number of mitochondrial diseases present aberrant mitochondrial morphology, including mitochondrial fragmentation or excessive mitochondrial fusion, which have an effect on mitochondrial function, leading to dysfunction of vital organs and tissues and accordingly threatening patients’ health and survival [3–5].

We first identified *SLC25A46* as a pathogenic gene related to mitochondrial function [6]. *SLC25A46* mutations can lead to highly fused mitochondria and decreased mitochondrial oxidative phosphorylation (OXPHOS). This result contradicts the traditional view that mitochondrial fusion is beneficial to the improvement of OXPHOS [7]. Therefore, from the perspective of sub-mitochondrial structure and mitophagy, it is more likely that damage to the sub-mitochondrial structure or an increase in mitophagy underly the mitochondrial dysfunction in the patients with *SLC25A46* mutations.

Over the past few decades, mitochondrial three-dimensional (3D) structure and the structure of mitochondrial cristae could only be observed by transmission electron microscopy (TEM). However, TEM cannot be applied to living cells and 3D observation, and is also time-consuming and expensive [8]. On the other hand, the confocal microscope is unable to visualize and quantitatively calculate the structure of sub-mitochondria [9]. 3D-structured illumination microscopy (SIM) is nanoscale microscopy which illuminates living cells by patterned excitation light, and then reconstructs the image in silico to achieve a doubling of the spatial resolution in all three dimensions [10]. With a spatial resolution of 100–120 nm, 3D-SIM was recently developed to observe and quantify mitochondrial morphology, sub-mitochondrial structure, mitophagy, mitochondrial dynamics, and the interaction of organelles [11–13]. Therefore, 3D-SIM, can accomplish multiple experimental purposes with a single technique by achieving the comprehensive observation of mitochondrial 3D morphology, sub-mitochondrial structure, mitophagy, and mitochondrial dynamics. In this paper, we have set out to take advantage of the live-cell nanoscope-3D-SIM to dynamically observe the mitochondrial and sub-mitochondrial morphology in the fibroblasts derived from the patients carrying biallelic mutations in *SLC25A46*. The results were consistent with the TEM by another group [14], suggesting that our 3D-SIM based analysis technique was reliable. Combined with the sub-mitochondrial structure identification/quantification and mitochondria-lysosome interaction quantification methods developed by our group [15–18], we found that the damage of mitochondrial cristae was the most probable cause of mitochondrial dysfunction in patients with *SLC25A46* mutations, and that the damaged mitochondrial cristae did not induce mitophagy. This study indicates 3D-SIM can be used to evaluate sub-mitochondrial structural damage in living cells and identify the pathology for patients with mitochondrial disease.

## Results

### Patient-derived *SLC25A46* mutant fibroblasts reveal abnormal mitochondrial functions

**Fig. 1:**
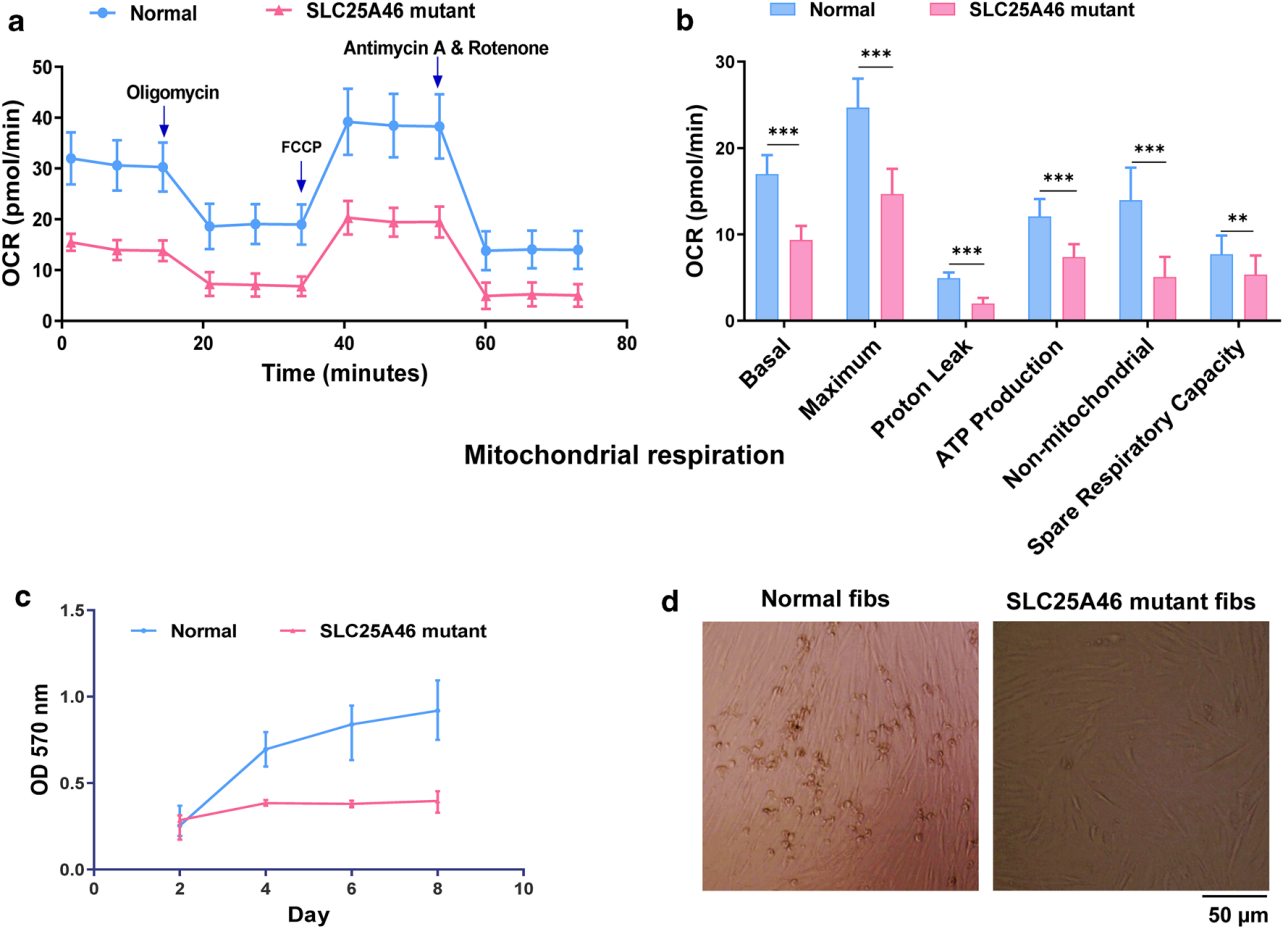
Comparative analysis of mitochondrial respiration and cell proliferation in human-derived normal and *SLC25A46* mutant fibroblasts. The oxygen consumption rate (OCR) (**a**, **b**) and the cell proliferation rate during one week after the seeding of the cells (**c**, **d**) are shown for human-derived normal and *SLC25A46* mutant fibroblasts. Data appear as mean ± SD; ***p < 0.001, **p < 0.01, as compared to normal fibroblasts

Sanger sequencing results showed a homozygous, missense point mutation (c.1005 A>T; p.Glu335Asp) in *SLC25A46* mutant fibroblasts (Additional file 1). To examine mitochondrial function of *SLC25A46* mutant fibroblasts, the mitochondrial respiration function was investigated by examining OCR under both basal conditions and drug-induced mitochondrial stress using the Seahorse assay. The OCR was found to be significantly decreased in patient-derived *SLC25A46* mutant fibroblasts compared to normal fibroblasts (Fig. 1a). After a detailed analysis, the basal respiration, oxygen consumption for adenosine triphosphate (ATP) production, maximum oxygen consumption capacity of mitochondria, proton-leaked oxygen consumption, non-mitochondrial respiration, and the spare respiratory capacity in patient-derived *SLC25A46* mutant fibroblasts were all lower than that of normal fibroblasts (Fig. 1b). This result is consistent with the previous finding that *SLC25A46* mutations cause decreased mitochondrial OXPHOS.

The 3-(4,5-dimethylthiazol-2-yl)-2,5-diphenyl tetrazolium bromide (MTT) assay reflects the metabolic ability of living cells by measuring the proliferation rates of cells. The results of this assay for the mutant and normal fibroblasts showed no apparent difference in the number of living cells between these two types of fibroblasts on day 2 after cell seeding. However, subsequent to this time point, the normal fibroblasts showed vigorous metabolism and rapid proliferation rate on day 4, day 6, and day 8 (Fig. 1c). Thus, the metabolic ability and cell proliferation rate of *SLC25A46* mutant fibroblasts were significantly lower than that of normal fibroblasts (Fig. 1c). The imaging results showed that the cell density of normal fibroblasts was close to 80–90% on day 8, while it only reached 40–50% in *SLC25A46* mutant fibroblasts (Fig. 1d). Our results suggest that *SLC25A46* mutations affect cell proliferation, probably through decreased mitochondrial respiration.

### Nanoscope-3D-SIM imaging system demonstrates mitochondrial hyper-fusion in the living patient-derived *SLC25A46* mutant fibroblasts

**Fig. 2:**
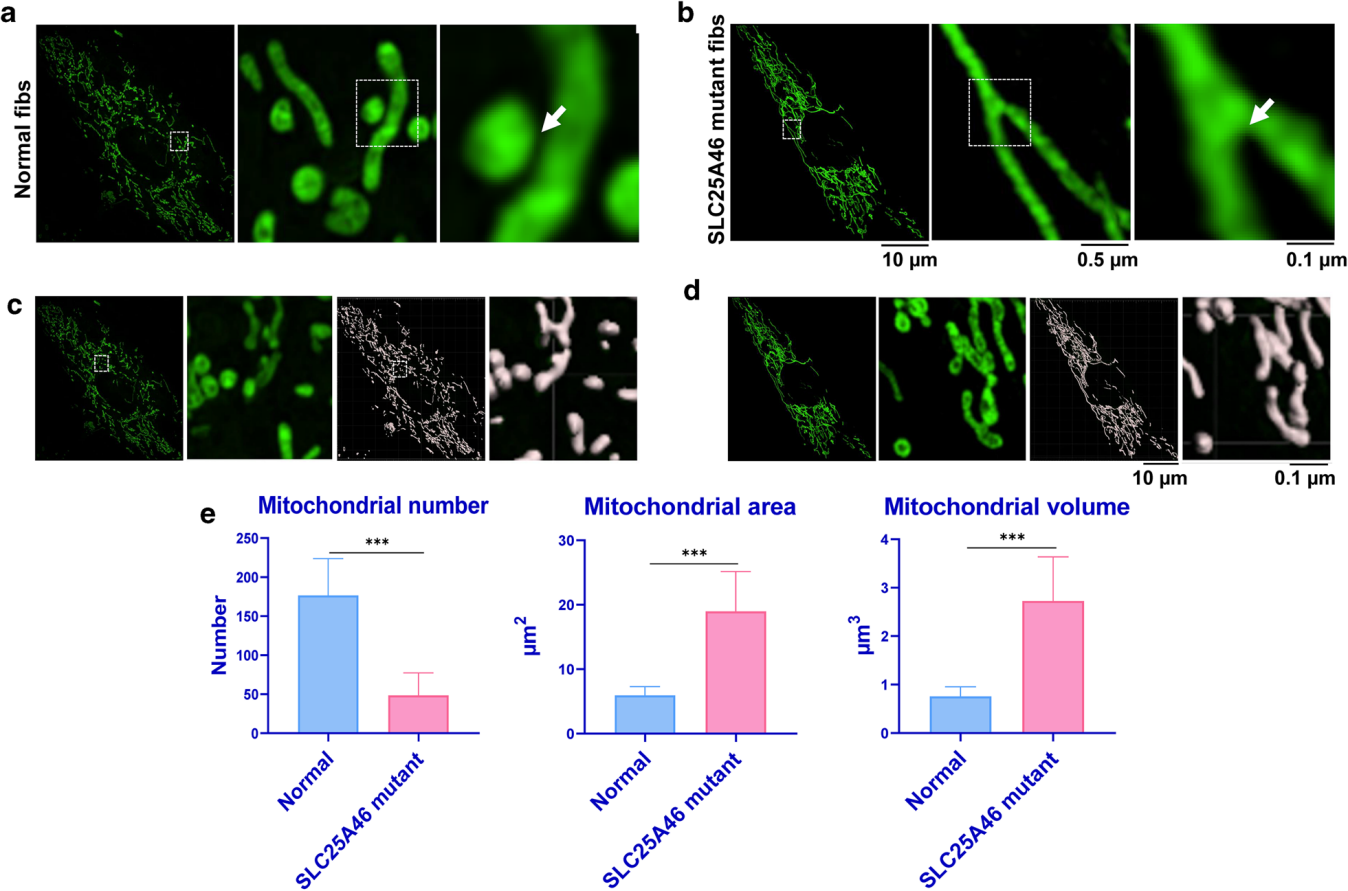
Comparative analysis of mitochondrial morphology in human-derived normal and *SLC25A46* mutant fibroblasts. The mitochondrial morphology is shown for human-derived normal (**a**) and *SLC25A46* mutant fibroblasts (**b**). Quantitative analysis of mitochondrial number was performed by Imaris (**c**, **d**). The results of the quantitative analysis of the mitochondrial area and volume by the Imaris software are also shown (**e**). Data are presented as mean ± SD (n = 8); ***p < 0.001, as compared to normal fibroblasts

The decreased metabolic ability of mutant fibroblasts suggested that the mitochondrial function in *SLC25A46* mutant fibroblasts has been disturbed. To examine whether this mutation causes any changes in mitochondrial morphology, we used a nanoscope-3D-SIM imaging approach to observe mitochondrial morphology in these two human cell lines. The images showed that the normal fibroblasts had round or medium length mitochondria (Fig. 2a), while the *SLC25A46* mutant fibroblasts showed slender, hyper-fused mitochondria (Fig. 2b). Imaris software (Nikon, Tokyo, Japan) was used to identify and analyze the mitochondrial morphology (Fig. 2c, d). The results showed that the number of mitochondria in *SLC25A46* mutant fibroblasts was significantly lower than what was observed in normal fibroblasts. In contrast, the average area and volume of mitochondria in mutant cells were significantly greater than those in normal fibroblasts (Fig. 2e). The comparative analysis of mitochondrial morphology showed aberrant hyper fusion of mitochondria in the patient-derived *SLC24A46* mutant fibroblasts.

### Severe damage of mitochondrial cristae in patient-derived *SLC25A46* mutant fibroblasts

Previously, mitochondrial fusion was considered to facilitate OXPHOS, and an increase of mitochondrial fusion was assumed to improve the mitochondrial OXPHOS level [7, 19]. Mediated mitochondrial fusion was therefore regarded as a new therapeutic target for mitochondrial diseases [20, 21]. However, our group found that the highly-fused mitochondria from *SLC25A46* mutant fibroblasts resulted in reduced OXPHOS [6] and our results here also confirmed that *SLC25A46* mutant fibroblasts have a low respiration as measured by SeaHorse (Fig. 1). What is the underlying pathogenesis for *SLC25A46* mutations? One possibility is that it is related to alterations in the cristae, one of the most critical structures of the inner mitochondrial membrane (IMM), which are deemed as the core of ATP production and mitochondrial respiratory function [22, 23]. Therefore, we decided to investigate whether structural defects of mitochondrial cristae lead to decreased OXPHOS.

**Fig. 3:**
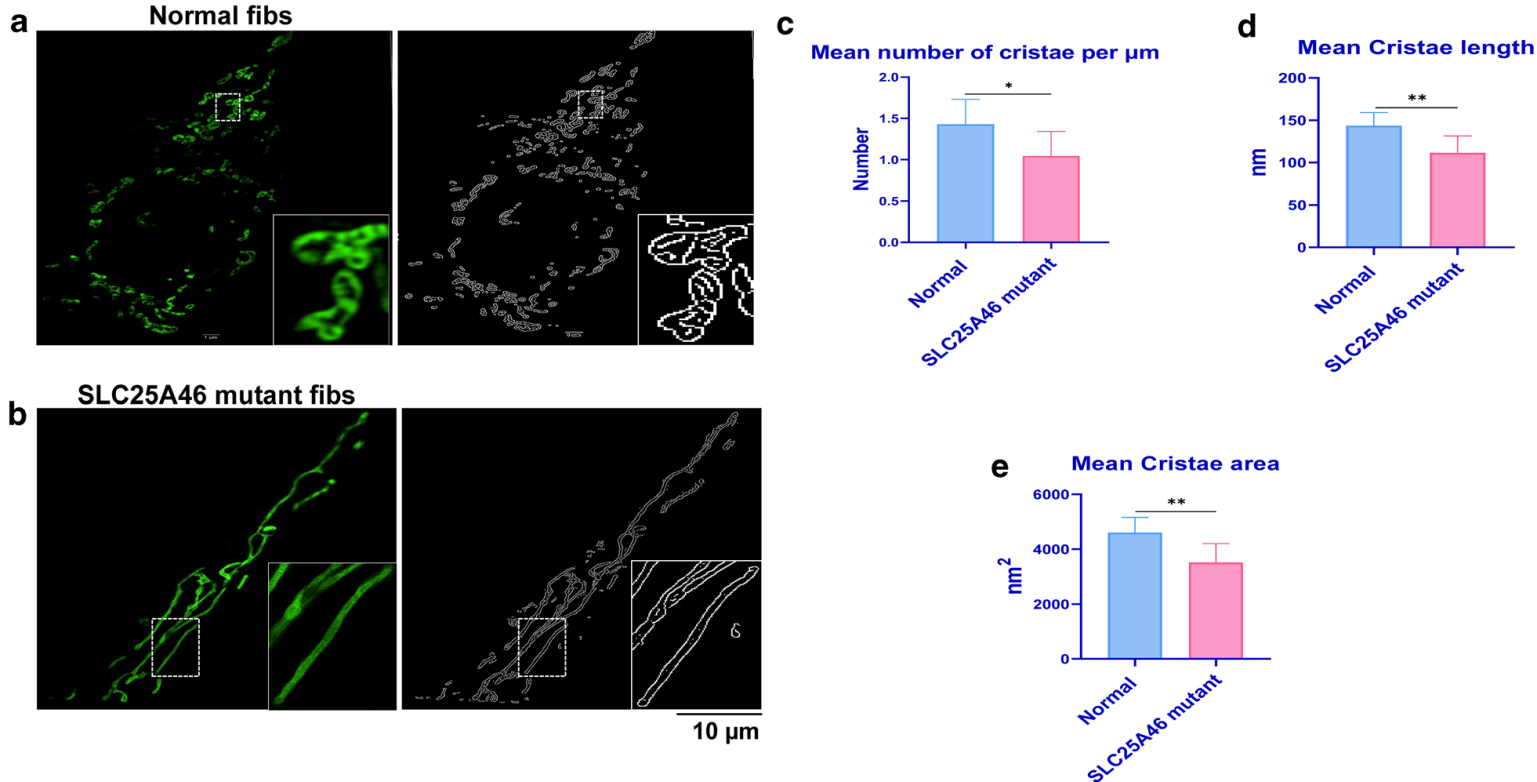
Cristae analysis of mitochondria in human-derived normal and *SLC25A46* mutant fibroblasts. Normal cristae (**a**) were observed in human-derived normal fibroblasts, while invisible cristae and a reduced number and length of cristae (**b**) were observed in *SLC25A46* mutant fibroblasts. Quantification analysis of cristae included the number of cristae per µm (**c**), cristae length (**d**), and cristae area (**e**). Data are present as mean ± SD (n = 8); **p < 0.01, *p < 0.05, as compared to normal fibroblasts

Using algorithm-based SIM imaging technology previously developed by our team [15], we first identified and extracted cristae, then quantitatively analyzed the mitochondrial cristae for human-derived normal and patient-derived *SLC25A46* mutant fibroblasts. The images showed that the mitochondrial cristae structure was visible and abundant in normal fibroblasts (Fig. 3a). In contrast, the cristae structure was damaged or even vanished in *SLC25A46* mutant fibroblasts (Fig. 3b). After quantification analysis, the mean cristae number (Fig. 3c), cristae length (Fig. 3d), and cristae area (Fig. 3e) of *SLC25A46* mutant fibroblasts all showed significantly lower values than those observed in normal fibroblasts.

### *SLC25A46* mutations do not significantly alter mitophagy in fibroblasts

**Fig. 4:**
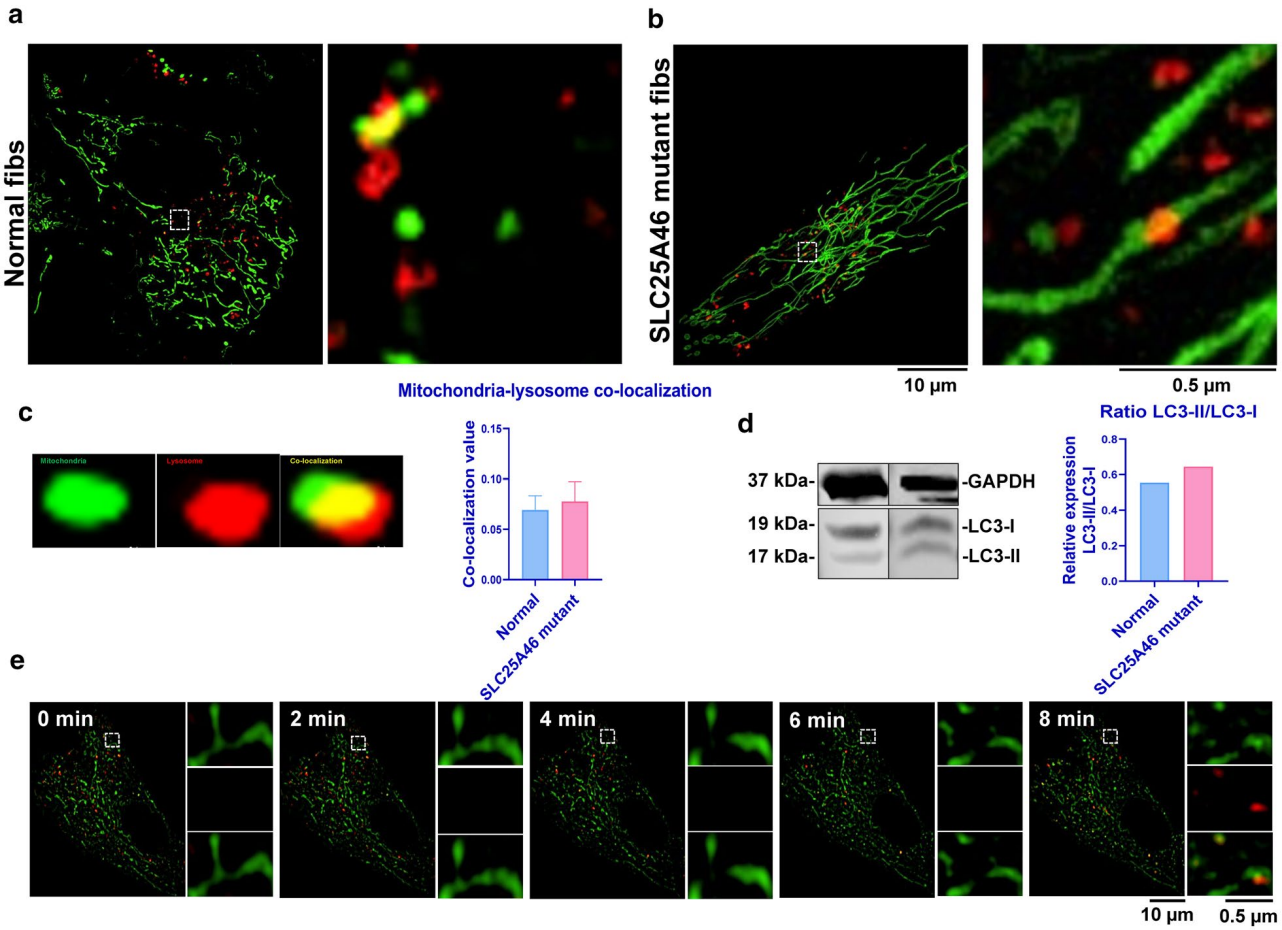
Mitophagy analysis in human-derived normal and *SLC25A46* mutant fibroblasts. The same trend of mitophagy is observed in the human-derived normal and *SLC25A46* mutant fibroblasts (**a**, **b**). This was confirmed by the mitochondria-lysosome co-localization value (**c**) and Western-blot (**d**). The mitochondrial dynamics and lysosome-mediated mitophagy could be clearly observed by time-lapse (**e**)

Mitophagy is the general process by which the cell removes severely damaged mitochondria, consequently achieving the purpose of “quality control” of mitochondria within living cells [24, 25]. We observed highly-fused mitochondria with severely damaged cristae structures in *SLC25A46* mutant fibroblasts. This raised the obvious question of whether or not these abnormal mitochondria induce mitophagy? Using the SIM image-based mitochondria-lysosome co-location analysis method in living cells [16], we can observe and quantify mitophagy in normal and *SLC25A46* mutant fibroblasts (Fig. 4a–c).

Our results demonstrate that only a low level of mitophagy occurs in both normal and mutant cell lines (Fig. 4a, b). After quantitative analysis, there was no statistically significant difference in the value of mitochondrial-lysosome co-location between the normal and *SLC25A46* mutant fibroblasts (Fig. 4c). Western Blot also confirmed that the values of the light chain 3-II (LC3-II)-II/LC3-I ratio were comparable between normal and *SLC25A46* mutant fibroblasts (Fig. 4d), which was consistent with the results of the SIM image-based analysis method. This result is confirmed in our 3D-SIM analysis by straightforwardly monitoring the mitochondrial dynamics and the mitochondria–lysosome interaction dynamics in human fibroblasts (Fig. 4e).

**Fig. 5:**
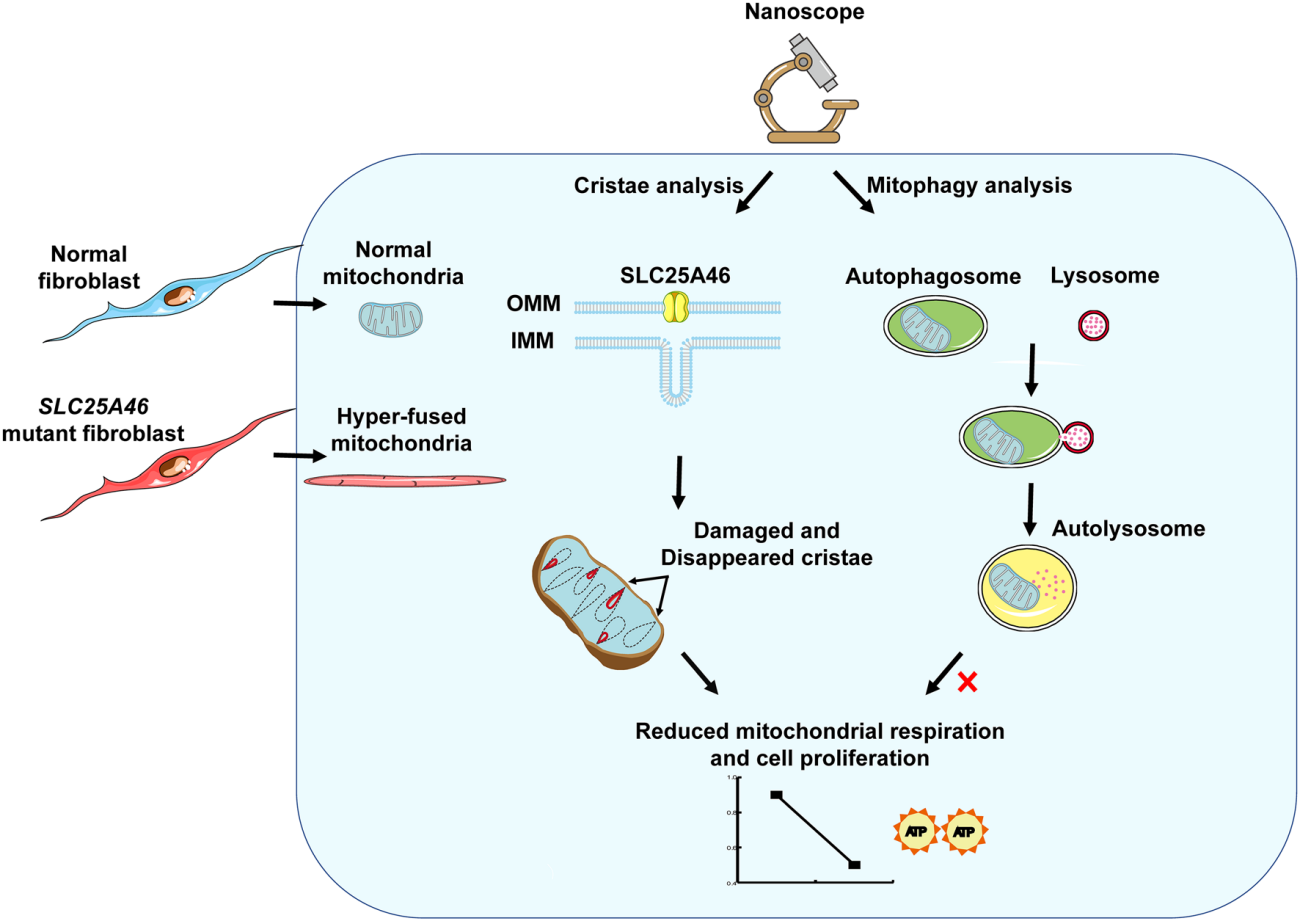
Summary of results for the examination of mitochondrial dysfunction using the nanoscope. The nanoscope can be used to closely analyze the structure of mitochondrial cristae, mitophagy, and mitochondrial dynamics in living cells, which is an extremely suitable application for the clinical analysis of the causes of mitochondrial dysfunction in patients with mitochondrial diseases

Our results showed that a combination of nanoscope with a quantification analysis strategy can not only be used to observe mitochondrial morphology, but also to detect and quantify damage to sub-mitochondrial structures, assess the extent of mitophagy, and monitor the dynamics of mitochondria and lysosomes (Fig. 5). This provides a novel means of observing and identifying pathology in patients with mitochondrial disease, which is critical to guide the development of the treatment.

## Discussion

*SLC25A46* is responsible for encoding a mitochondrial solute carrier protein [26]. We previously identified SLC25A46 is the human homolog of Ugo1, a protein of *Saccharomyces cerevisiae* and located in the mitochondrial outer membrane and involved in mitochondrial fusion [6, 27, 28]. So far, SLC25A46 has been found to be associated with various human diseases. Homozygous or compound heterozygous mutations of *SLC25A46* led to a range of clinical syndromes, with the clinical feature of optic atrophy, cerebellar atrophy, progressive myoclonic ataxia, axonal peripheral neuropathy, autosomal recessive cerebellar ataxias (ARCA), lethal congenital pontocerebellar hypoplasia, and even Parkinson’s disease [6, 26, 29–34]. Mice with *Slc25a46* dysfunction developed severe motor impairment, optic atrophy, and developmental defects of the nervous system, as well as premature death [35–37].

Currently, SLC25A46 is believed to affect mitochondrial dynamics due to the interaction with dominant optic atrophy 1 (OPA1) and Mitofusin-2 (MFN2) [14, 38]. The hyper-fused mitochondria and reduced mitochondrial respiratory function presented in patient-derived *SLC25A46* mutant fibroblasts have also been confirmed by this study, as well as previous studies, which were supposed to be the pathogenic mechanism of a series of neurological diseases [39]. The MTT assay results from this study also strengthened the idea that the metabolic capacity of *SLC25A46* mutant fibroblasts is significantly lower than that of control cells. However, there exists a contradiction about the morphology of highly-fused mitochondria and the decline of mitochondrial function. Traditionally, mitochondrial fusion has been verified to be vital for maintaining mtDNA stability and improving the tolerance of cells to high mtDNA mutations [40, 41]. At the same time, mitochondrial fusion is also a protective factor for maintaining normal mitochondrial respiration function. The absence of mitochondrial fusion in the cerebellum has also been shown to result in a malformed mitochondrial distribution and function [42]. Moreover, mitochondrial fusion is required to support the normal development of embryos [3].

Why then do the *SLC25A46* mutant cells examined in our study show mitochondrial hyper-fusion but a decrease in mitochondrial respiratory function? The respiratory function of mitochondria is a series of oxidation-reduction reactions mediated by multiple complexes located on the mitochondrial inner cristae, which eventually produce ATP and provide energy for the tissues and cells in living organisms [43, 44]. From this viewpoint, we hypothesized that *SLC25A46* mutation causes structural abnormalities of cristae in highly fused mitochondria, consequently affecting mitochondrial respiratory function. Based on the identification and quantification method of mitochondrial cristae developed by our group, we analyzed the mitochondrial internal cristae of patient-derived *SLC25A46* mutant fibroblasts and human-derived normal fibroblasts. Our results showed that, compared with normal mitochondria, the number of mitochondrial cristae decreased, the length of cristae shortened, and the area of cristae was reduced in the *SLC25A46* mutant fibroblasts. We even observed the disappearance of cristae in some mitochondria. Therefore, we posited that the structure of mitochondrial cristae in the mutant cells was damaged, which therefore affected the mitochondrial respiratory function, as reflected by decreased aerobic respiration, reduced ATP generation, and decreased metabolic capacity. Researchers have suggested that SLC25A46 plays a vital role in the interaction between the major structural proteins of the mitochondrial outer membrane and the mitochondrial cristae, and it is crucial for maintaining the structure and stability of the mitochondrial cristae. Immunoblot analysis revealed that MICOS complex subunit 60 (MIC60) and MICOS complex subunit 19 (MIC19)—two critical proteins of mitochondrial contact site and cristae organizing system (MICOS) complex—were clearly decreased in patient-derived *SLC25A46* mutant fibroblasts. An immunoprecipitation experiment showed that SLC25A46 co-immunoprecipitated with MIC60, MIC19, OPA1 (located on IMM), MFN1 and MFN2 (located on OMM) [14]. MICOS complex, especially MIC60 and MIC19, is a crucial factor in cristae biogenesis [45, 46]. Therefore, SLC25A46 is believed to be not only involved in maintaining the stability of OMM, but also an essential protein in the interaction and communication between the OMM and IMM, as well as the formation and maintenance of mitochondrial cristae [14]. Our group as well as others have used TEM and observed the significantly decreased cristae number and length from patient-derived mitochondria [14]. Our results in 3D SIM are consistent with the findings from TEM analysis, verifying the reliability of our nanoscope-based method.

Mitophagy is known to be an autophagy process that selectively eliminates excess or damaged mitochondria. It plays a vital role in regulating the number of mitochondria in cells and maintaining mitochondrial quality control. It is involved in many physiological and pathological processes [47, 48]. Severe injury of mitochondrial cristae can induce mitophagy as well [49, 50]. Therefore, we also hypothesized that the damaged mitochondrial cristae would increase the rate of mitophagy in *SLC25A46* mutant fibroblasts. We monitored the dynamic changes of mitochondrial and lysosomal behavior in *SLC25A46* mutant fibroblasts in real-time. We observed a contact and co-localization phenomenon between lysosomes and mitochondria after mitochondrial fragmentation in *SLC25A46* mutant fibroblasts. However, using the SIM image-based mitophagy quantification method, we determined that the overall tendency of mitophagy in the *SLC25A46* mutant fibroblasts was not statistically different from that in normal fibroblasts, although mitophagy did occur in some mitochondria in the *SLC25A46* mutant fibroblasts. Consequently, although the mitochondrial cristae were severely damaged in the *SLC25A46* mutant fibroblasts, the damaged cristae alone did not appear to induce mitophagy in this particular cell line. Why then did the damage to mitochondrial cristae not stimulate mitophagy? Is it because the mitochondrial cristae damage alone is not enough to trigger mitophagy, or are there defects in the mitophagy process in *SLC25A46* mutant fibroblasts? According to the characteristics of the mitophagy process, the precondition for mitophagy is the membrane permeability change after mitochondrial damage. This leads to mitochondrial depolarization and induces the activation of mitophagy-related proteins. Subsequently, damaged mitochondria are wrapped by the early autophagosomes, and then mitophagosomes are formed. Therefore, we will likely focus on the following questions in the near future: (1) Is there any change in mitochondrial membrane potential in *SLC25A46* mutant fibroblasts? We can thus determine whether mitochondrial damage is sufficient to induce mitophagy. (2) Is there any alteration in the expression of mitophagy-related proteins (such as PINK1, Parkin, MUL1, etc.) in *SLC25A46* mutant fibroblasts? We can also explore this question by studying whether there are mitochondrial autophagy deficiencies in the mutant fibroblasts. Currently, no studies have reported the mitophagy status of *SLC25A46* mutant cells.

## Conclusions

Overall, this study shows that nanoscope-based imaging is a reliable method for analyzing the sub-mitochondrial morphology, mitophagy and mitochondrial dynamics in living cells. This method may be particularly valuable for the quick evaluation of the pathogenesis of mitochondrial morphological abnormalities. Utilizing this tool, we were able to show that severely damaged mitochondrial cristae may be the predominant cause of reduced mitochondrial respiratory dysfunction in *SLC25A46* mutant fibroblasts. This approach also showed that damaged mitochondrial cristae do not appear to be sufficient to induce a significant increase in mitophagy in this particular condition.

The significance of this study is that is will likely be possible to apply this method to a wide range of mitochondrial diseases by examining the sub-mitochondrial structure of living cells. Moreover, the 3D-SIM can image up to 4 fluorecent channels, which can also be used for simultaneously monitoring the dynamics of fluorophore-labeled proteins. Another advantage of this method is that we are able to identify the mitochondrial 3D morphology, mitophagy, and mitochondrial dynamics using one technique. Therefore, it is exceptionally suited for those patients with mitochondrial diseases related to morphological disruption. The operation of the device is simple and rapid, which is valuable for the quick assessment of mitochondrial morphological abnormalities and the identification of the potential pathology, and may eventually help facilitate the screening of lead compounds for the treatment of such disorders.

## Methods

### Cell culture

The human-derived normal fibroblasts and patient-derived *SLC25A46* mutant (c.1005 A>T, p.Glu335Asp) fibroblasts cell lines were acquired after informed consent was obtained from the patients. The cells were cultured in Dulbecco’s modified Eagle’s medium (DMEM) medium (Gibco, Thermo Fisher Scientific, USA) with 10% Fetal Bovine Serum (FBS) (Gibco, Thermo Fisher Scientific, USA) and 100 units/ml Anti-Anti (containing streptomycin and penicillin) (Gibco, Thermo Fisher Scientific, USA) and incubated in a 5% CO_2_, 37 °C and 100% humidity incubator.

### Nanoscope—3D-SIM imaging

The cells were seeded in a glass-bottom culture dish (MatTek Life Sciences, USA) and cultured for 24 h in 2 ml DMEM containing 10% FBS and 100 units/ml Anti-Anti. Before imaging, cells were first washed three times with a pre-warmed DMEM medium and then were incubated in a DMEM medium containing 100 nM Mito-Tracker Green (Invitrogen, USA) for half an hour. Cells for mitophagy analysis were co-incubated in DMEM medium containing 100 nM Mito-Tracker Green (Invitrogen, USA) and Lyso-Tracker Red (Invitrogen, USA) for half an hour. Cells were then washed three times with DMEM. The stained cells were photographed using the 3D-structure illumination microscope (Nikon, Tokyo, Japan).

### Western blot

Protease inhibitor cocktail (Sigma, USA) and 2× RIPA lysis and extraction buffer (Thermofisher Scientific, USA) were added to the centrifuged cell pellets, and were then sonicated for 5 min each time, three times in total. The protein concentration was measured using the Pierce BCA Protein Assay Kit (Thermofisher Scientific, USA). 30 µg protein for each sample and 4× NuPAGE LDS Sample Buffer (Thermofisher Scientific, USA) were mixed at 4:1 ratio and denatured at 95 °C for 5 min, and then separated in 4–12% Bis–Tris gel (Invitrogen, USA). The gel was transferred onto a PVDF membrane (Invitrogen, USA) through the iBlot 2 gel transfer device (Life Technologies, USA). The transferred PVDF membrane was placed in the Intercept Blocking Buffer (LI-COR Biosciences, USA) for 45 min, and then incubated overnight in the primary antibody, rabbit anti-LC3B (cell signaling technology, USA) diluted in the blocking buffer at a ratio of 1:200 with Tween 20 diluted in the blocking buffer at a ratio of 1:1000. Rabbit anti-GAPDH (cell signaling technology, USA) was also diluted in the blocking buffer at a ratio of 1:2000 and set as the loading control. The next day, the PVDF membrane was washed for 10 min each time, three times in total. Then, the membrane was incubated in the secondary antibody, IRDye 800CW Goat anti-Rabbit IgG (LI-COR Biosciences, USA), for 120 min. The bands were detected by the LI-COR Odyssey Clx Imaging System (LI-COR Biosciences, Lincoln, NE).

### Sanger sequencing for mutation detection

To detect the point mutation of *SLC25A46* in human-derived normal and patient-derived fibroblasts, genomic DNA was extracted using DNeasy Blood & Tissue Kit (Qiagen, USA). PCR products of 186 bp in length were amplified using GoTag master mixes (Promega, USA). The following primer set was used for the amplification: Forward: TGCCAGTCTTTGTTCTGACG and Reverse: CCAAACACTCCTTCCTCCTG. The reactions were performed following the thermal cycling program: 95 °C for 2 min, followed by 30 cycles of 95 °C for 30 s, 56 °C for 30 s, and 72 °C for 30 s. A final extension step was then performed at 72 °C for 4 min.

### Oxygen consumption rate (OCR) measurement

Human-derived normal and patient-derived *SLC25A46* mutant cells were seeded at a density of 1.0 × 10^4^ cells/well with DMEM supplemented with 10% FBS in XFe96 cell culture plates (Agilent Technologies, USA). After incubation for 24 h, the DMEM medium was removed and changed with the warmed XF DMEM Medium supplemented with 1 mM sodium pyruvate, 10 mM glucose and 2 mM l-glutamine at pH 7.4. All cells were treated with 1 µM oligomycin A, 1 µM FCCP, and 500 nM rotenone/antimycin A. The OCRs of the cells was assessed by using the XF Cell Mito Stress Test Kit (Agilent Technologies, USA). The Seahorse XF96 analyzer (Agilent Technologies, USA) was used for OCR measurement.

### Cell proliferation rate measurement (MTT assay)

Human-derived normal and patient-derived *SLC25A46* mutant fibroblasts were seeded in 96 well plates (Corning, USA) at a density of 3.0 × 10^3^ cells/well with DMEM supplemented with 10% FBS and incubated at 37 °C, 5% CO_2_. 10 µl of MTT solution (Roche, USA) was added to 100ul culture medium in each well at a final concentration of 0.5 mg/ml. The following process was implemented according to the manual provided by the kit. The absorbance was detected at 570 nm by the microplate reader (BioTek, USA).

### Statistical analysis

Graphpad Prism 7 software was used to display data. Independent-samples T-test was used for statistical analysis. * was defined as *P* < 0.05, ** as *P* < 0.01, *** as *P* < 0.001, and **** as *P* < 0.0001.

## Supplementary Information

**Additional file 1.** Sanger sequencing of the mutant variant of human-derived normal and *SLC25A46* mutant fibroblasts. Sanger sequencing results showed a homozygous, missense point mutation (c.1005A>T; p.Glu335Asp) in *SLC25A46* mutant fibroblasts (B). The normal variant at this site is also shown for comparison (A).

## Data Availability

The datasets used and/or analysed during the current study are available from the corresponding author on reasonable request.

## Abbreviations

OXPHOS: Oxidative phosphorylation
nDNA: Nuclear DNA
mtDNA: Mitochondrial DNA
3D: Three dimensional
SIM: Structured illumination microscopy
ATP: Adenosine triphosphate
MTT: 3-(4,5-Dimethylthiazol-2-yl)-2,5-diphenyl tetrazolium bromide
LC3: Light chain 3
OCR: Oxygen consumption rate
IMM: Inner mitochondrial membrane
OMM: Outer mitochondrial membrane
ARCA: Autosomal recessive cerebellar ataxias
OPA1: Dominant optic atrophy 1
MFN2: Mitofusin-2
MIC60: MICOS complex subunit 60
MIC19: MICOS complex subunit 19
MICOS: Mitochondrial contact site and cristae organizing system
DMEM: Dulbecco’s modified Eagle’s medium
FBS: Fetal bovine serum
